# Application of multi-indexing approach within a GIS framework to investigate the quality and contamination of ground water in Barisal sadar, Bangladesh

**DOI:** 10.1016/j.heliyon.2025.e42262

**Published:** 2025-01-27

**Authors:** Md. Numan Hossain, M. Farhad Howladar, Sohag Ahammed, Md Rezwanul Haque, Majedul Islam Khan, Muyeed Hasan, Tayabur Rashid Chowdhury, Alamgir Hosain

**Affiliations:** a Department of Petroleum and Mining Engineering, Shahjalal University of Science and Technology, Sylhet, 3114, Bangladesh; b Department of Forest Policy and Management, Bangabandhu Sheikh Mujibur Rahman Agricultural University, Gazipur, 1706, Bangladesh; c Department of Geography and Environment, Shahjalal University of Science and Technology, Sylhet, 3114, Bangladesh; d Department of Coastal Studies Disaster Management, University of Barisal, Barisal, Bangladesh

**Keywords:** GIS framework, Groundwater contamination, Health risk assessment, Potential ecological risk, Principle component analysis (PCA), Water quality index, Multi-indexing approach

## Abstract

Groundwater quality and contamination pose significant challenges in coastal regions such as Barisal Sadar, Bangladesh, where dependency on groundwater is crucial for potable and agricultural uses. This study employed a multi-indexing approach inside a GIS framework to assess water quality, contamination scenarios, and health risks. It evaluates the physicochemical properties of groundwater samples, including heavy metals, to identify potential issues. The results reveal that water's pH is slightly alkaline, with moderate to high levels of turbidity and hardness. The mean electrical conductivity is 1522 μS/cm, and most regions are above the threshold value. Total dissolved solids (TDS), chloride, and NaCl were found in elevated amounts, indicating water's impact on salinity. Heavy metal concentrations occasionally exceed permissible limits, indicating potential health hazards. The individual contamination indices range from low to high risk, whereas the weighted index signifies low to medium risk. The possible ecological risk was obtained within acceptable ranges except for some samples. The noncarcinogenic health risk remained below acceptable ranges (<1) throughout this investigation. In some instances, the carcinogenic risk of Cd and Ni was more significant than the international safe limit (1E-04). The water quality index indicates that 55 % of samples are rated excellent, 10 % moderate, 15 % poor, and 20 % very poor. The significant positive correlations among EC, TDS, TH, turbidity, salinity, NaCl, chloride, and calcium ions as per correlogram, principal component analysis (PCA) biplot, and heat map clustering, indicating similar sources of origin. Notwithstanding its constraints, this study represented one of the initial attempts in Barishal Sadar to investigate groundwater using numerous indices and GIS frameworks. In the end, policymakers can utilize this study's findings to monitor and control groundwater around the study area.

## Highlights

• Multiple contamination indices indicates low to moderate pollution around study area.
• The noncarcinogenic health risk below acceptable ranges (<1).
• The carcinogenic risk of Cd and Ni was significant than safe limit for some instances.
• About 55 %, 10 %, 15 %, and 20 % of samples are categorized as excellent, moderate, poor and very poor conditions as per WQI.
• Source apportionment indicates salt water instruction may affect on quality parameters.

## Graphical abstract

**Figure:**
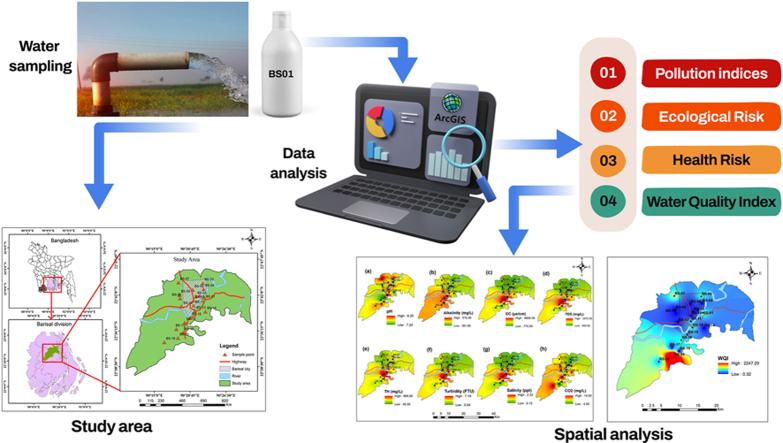

## 1 Introduction

The understanding of good water quality is essential in all aspects of the life cycle, encompassing societal prosperity, economic development, and human well-being [[1], [2], [3]]. The deprivation of water may result in the death of living organism compared to the absence of food [4]. Access to safe and high-quality water is considered a fundamental human right [5]. Nonetheless, inadequate water quality is a pressing issue in contemporary times, as water is significantly contaminated due to escalating patterns of human growth [6,7]. According to statistical data, around 1.1 billion people globally lack access to potable water [8]. Moreover, 80 % of diseases prevalent in underdeveloped nations are waterborne [4,9], indicating the significance of quality water for safe consumption [10].

Water quality denotes the intrinsic attributes of water, encompassing physical, chemical, and biological qualities [11]. The decline in water quality may arise from several forms of pollution originating from industrial, agricultural, and urban activities [[10], [11], [12]], posing ecological and environmental risks, as well as health hazards for consumers [13]. Numerous techniques around the world are employed to ascertain the appropriateness, classification, and categorization of water resources [14], multi indexing approaches is one of them [15,16]. This powerful technique combines multiple chemical, physical, and biological markers into a single value with improved precision and sensitivity to measure the health of water body [17]. Different indicators, including water quality index (WQI), pollution indices (PI), weighted contamination index (WCI), potential ecological risk index (PER), health risk index (HR), and so on are widely used for this purposes [3,6,9,15]. WQI provides a comprehensive assessment of water quality incorporating physiochemical parameters of water [14,18]. PI is utilized to assess the contamination scenario, while WCI represents an overall pollution scenario amalgamated using numerous contamination indices [3]. In addition, PER evaluates potential ecological consequences from heavy metals in aquatic environments [19]; health risk (HR) examines the carcinogenic and non-carcinogenic hazards linked to toxic elements present in water [20,21]. Moreover, inferential and descriptive statistics are employed to identify specific sources of water pollution, which may encompass both natural processes and human activities [22,23]. Geographic Information Systems (GIS) are effective tools for water quality monitoring by collecting, analyzing, and managing spatial data [4]. This framework employs an analytical algorithm using geoprocessing functions to generate interactive maps from preexisting data sets. The spatial analyst extension of ArcGIS offers a variety of sophisticated interpolation capabilities, widely utilized for global water quality monitoring [24].

Numerous researchers around the world have conducted studies on drinking water quality assessment applying multivariate statistical approaches within GIS framework, specifically in Afghanistan [25], Brazil [26], China [27], Colombia [28], Ethiopia [29], India [24], Iran [30], Nigeria [31], Pakistan [32], Yemen [33], and so on [34,35]. A multitude of studies on drinking water quality monitoring have been conducted in several regions of Bangladesh, specially Barisal [36], Chattogram [37], Dhaka [38], Khulna [39,40], Jamalpur [41], Rajshahi [42], Rangpur [43], and Sylhet [44,45]. The researchers highlighted water quality and contamination scenarios from various perspectives, involving hadrochemical features of groundwater [39], seasonal variations in water quality [46], ecological and environmental risks [21,22], human health implications [47], and sources of groundwater pollution [45,48]. In Bangladesh, although water chemistry and degradation study are conducted sporadically, the investigated region has not been assessed for drinking water quality using multi-indexing approaches inside a GIS framework. Barishal is in the southern coastal region of Bangladesh, where saltwater intrusion into the groundwater table is a major concern [49]. Bangladesh water development board (BWDB) initiated a project to study surface and groundwater resources by mathematical modeling and assessing groundwater level changes and hydro geochemistry between 2012 and 2014, but later it went off [49]. Therefore, there was an absence of systematic investigation to examine the hydrochemistry of the Barisal region in relation to public health hazards and drinking water quality. By considering these factors this study was designed. The present study aims to (1) quantify the physicochemical parameters of drinking water along with spatial interpolation, (2) demonstrate contamination scenario of water samples using multiple indices, (3) assess potential ecological risk, health risk, and drinking water quality, and (4) evaluate source apportionment of water parameters around study area. At the end, the outcomes of the research will be assets for the proper authority of the studied areas. Additionally, the policymaker can utilize the results to implement the requisite measures to effectively manage the quality of potable water around study area.

## 2 Materials and methods

### 2.1 Area of interest

Barisal Sadar is in the Barisal district, a southern coastal portion of Bangladesh. It lies between 22°38′ and 22°45′ north latitudes and 90°18′ and 90°23′ east longitudes and covers an area of 24.91 square kilometers. Kaunia and Airport Thanas bound this region to the north, Bandar Thana to the south, Airport Thana and Kotwali Model Thana to the east, and Naclcity Upazila to the west [50]. In the research region, around 330,000 individuals reside, with their drinking water sourced from tube wells (77.37 %), ponds (4.19 %), taps (15.62 %), and other sources (2.82 %) [50]. The Kirtonkhola River runs through the city. Aside from domestic use, it is also an important source of irrigation water. Indeed, the river quality directly affects the drinking water in the respective areas.

The geology of this area affects groundwater potential and induces vertical and seasonal hydrogeochemical variations [49]. The studied area mainly comprises substantial Quaternary deposits, forming the uppermost shallow aquifers. The tertiary deposit, notable for its substantial groundwater development potential, comprises sandstone, argillaceous materials, pebble-bearing grits, variegated clays with lignite seams, and pebble beds, which form the principal and deeper aquifers [49]. Water-bearing strata deeper than 150–200 m are being utilized in coastal regions for urban water supply and rural drinking water; however, substantial extraction is prohibited due to the potential risk of seawater intrusion or leaking from the top aquifer [49,51].

### 2.2 Water sampling and analysis procedure

**Fig. 1:**
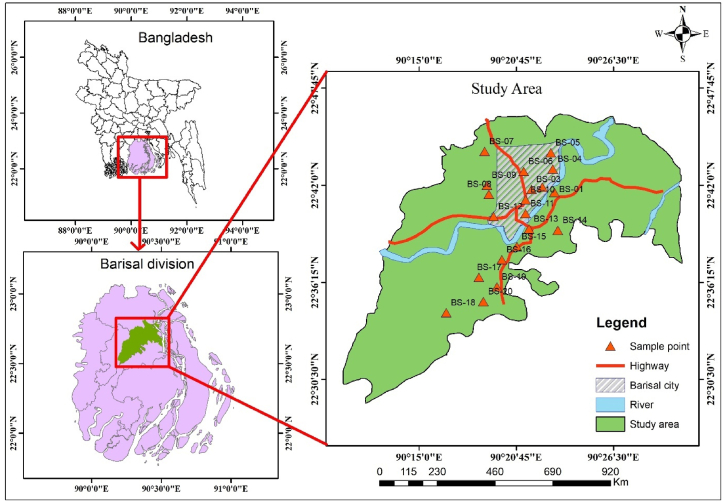
Base Map and sampling locations in the study area.

Water sampling was conducted around Barishal Sadar area following standard procedures [52]. A total of twenty tube-well water samples (depth typically 60–220 m) were obtained throughout the winter season for this study (Fig. 1). In winter, the limited rainfall in Bangladesh helps to prevent the dilution effect of water parameters and so yields superior results [53]. The samples were collected in 2-liter capacity clean, dry and sterilized plastic bottles. Each sample was properly leveled, the geological coordinates of the sampling sites were recorded, and in situ measurements of various water parameters (temperature, pH, electrical conductivity (EC), total dissolved solids (TDS), salinity, oxidation-reduction potential (ORP)) were performed using a portable water multi-parameter (HANNA HI 98194). Conversely, the turbidity of water was assessed with a turbidity meter (HI 93703). Calibration of each portable meter was completed with standard solutions as per the directives in their individual manuals [3]. After that, samples were brought to the laboratory for further analysis. The amount of carbon-di-oxide, salinity, NaCl, Cl^−^, total hardness (TH), and Ca^2+^ were measured in the laboratory of Petroleum and Mining Engineering, SUST, Sylhet by using different titrimetric methods. The laboratory analysis was performed following standard procedures of water and wastewater analysis developed by the American Public Health Association (APHA) [54]. The heavy metals, including Cd, Cu, Ni, and Pb, present in water were determined using an atomic absorption spectrophotometer (Model: AA-7000, Shimadzu Corporation, Japan) at Soil Resources Development Institute, Sylhet. In case of trace elements including Ni, Pb, Cd, Cu, Zn analysis, the National Institute of Scientific and Technology, USA proposed reference materials SRM-1640 and SRM-1643 have been employed to maintain the standard of analysis. Moreover, the accuracy and precision analysis were done to confirm the reliability of the data and calculation presented in the research. Following the laboratory study, statistical analysis and multiple indices of water parameter were conducted using a variety of software applications.

### 2.3 Contamination scenario analysis

The contamination scenario in drinking water around the Barishal Sadar area was evaluated using multiple indices. These include the total contamination index (TCI), modified contamination index (MCI), pollution load index (PLI), Nemerrow pollution index (NPI), metal pollution index (MPI), and heavy metal evaluation index (HEI). The contamination factor (CF) was initially assessed to derive additional pollution indicators. Equation (1) was employed to quantify CFi utilizing water parameter concentrations (Mi) and standard concentrations (Si). Moreover, summing the individual contamination is used to evaluate the total contamination index (TCI), presented in Equation (2). This study utilized the Bangladesh water quality standard value (BDWS) for Si, as detailed in Table 2.

(1) $${CF}_{i}=\frac{Mi}{Si}$$

(2) $$TCI=\sum_{i=1}^{i=n}CF$$

In this study, the modified contamination index (MCI) was evaluated by dividing the arithmetic mean of CF to the total number of sample (n), displayed by equation (3). This index provides an overall view of contamination by averaging the CFs, resulting in a more straightforward assessment of water quality.

(3) $$MCI=\frac{\sum_{i=1}^{i=n}CF}{n}$$

**Table 1:** Different indices and respective pollution levels are used in this study.

Indices	Pollution level					References
	Very low	Low	Medium	High	Very high	
TCI	–	TCI <8	8 ≤ TCI <16	16 ≤ TCI <32	TCI >32	[55]
MCI	MCI <1.5	1.5 ≤ MCI <2	2 ≤ MCI <4	4 ≤ MCI <8	MCI >16	[56]
PLI	–	PLI <0.5	0.5≤ PLI <1	PLI >1	–	[57]
NPI	NPI <1	1< NPI <2.5	2.5< NPI <7	NPI >7	–	[58]
MPI	MPI <0.3	0.3≤ MPI <1	1≤ MPI <2.5	2.5≤ MPI <7	MPI >7	[59]
Aw	1	2	3	4	5	[3]
WCI	WCI <0.07	0.07≤ WCI<0.2	0.2≤ WCI<0.4	0.4≤ WCI<0.67	WCI ≥0.67	[3]

**Table 2:** Understanding drinking water quality according to descriptive analysis around study areas.

This Study			Water quality standard				Percentage of samples within standards				
Parameters∗	Range∗	Mean ± St. Dev∗	WHO∗	USEPA∗	ISDW∗	BDWS∗	WHO∗	USEPA∗	ISDW∗	BDWS∗	Mean∗
pH	7.24–8.2	7.86 ± 0.26	6.5–8.5	6.5–8.5	6.5–8.5	6.5–8.5	100	100	100	100	100
Alkalinity	361.9–515	452.1 ± 43.5	200–600	30–400^1^	200	20–200	100	15	–	–	58
Free CO_2_	4–14	8.85 ± 2.86	10	–	–	N/A	75	100	100	100	94
EC	770–4800	1522 ± 946.8	250	50–1500^**2**^	–	50–1500	0	70	100	70	60
ORP	−64.3 to −21.5	−42.13 ± 10.62	–	–	–		–	–	–	–	–
Salinity	0.15–2.33	0.52 ± 0.50	–	<1^3^	<1^4^	0.6^5^	–	90	90	75	85
TDS	450.6–3072	971.9 ± 607.8	600–1000	500	500	1000	75	5	5	75	40
TH	45–665	151.9 ± 128.1	300	200^1^	300	200–500	95	90	95	90	93
Turbidity	0.04–7.19	1.17 ± 1.55	4	5	5	10	95	95	95	100	96
NaCl	140.3–2127.2	472.6 ± 459.7	200–300	500^2^	–	600^6^	40	75	–	75	64
Ca^2+^	18.04–264.5	60.78 ± 50.88	75	–	75	75	100	–	100	100	100
Cl^−^	85.1–1290.4	472.6 ± 278.6	250	250	250	150–600	85	100	85	85	89
Cd	0.006–0.129	0.026 ± 0.038	0.003	0.005	0.003	0.005	70	70	70	95	76
Cu	0.003–0.02	0.008 ± 0.005	2.00	1	0.005	1.00	55	55	55	55	55
Ni	0.02–0.24	0.084 ± 0.089	0.07	–	0.02	0.1	100	–	75	100	94
Pb	0.09–0.271	0.213 ± 0.055	0.01	0.015	0.1	0.05	65	100	60	70	74

Parameters∗ = Parameters unit used as EC (μS/cm), ORP (mV) salinity (ppt), turbidity (FTU), and others (mg/L) excepting pH. Range∗ = minimum-maximum value, St. Dev∗ = Standard deviation, WHO∗ = world health organization drinking water standards [76], USEPA∗ = United states environmental protection agency water quality standards [80], ISDW∗ = Indian Standards for drinking water quality [81]. BDWS∗ = Bangladeshi water quality standards according to environmental conservation rules [82], Mean∗ = mean indicated percentages of samples exceeding all mentioned standards (1 = [83]; 2 = [84]; 3 = [85]; 4 = [86]; 5 = [37]; 6 = [68]).

The pollution load index (PLI) is used to determine the overall contamination level by considering the cumulative effect of multiple contaminants. It is evaluated as the geometric mean of the contamination factor (Equation (4)).

(4) $$PLI=\sqrt[{n}]{nCF}$$

The Nemerrow pollution index (NPI) provides an in-depth pollution assessment by considering mean and maximal CF. Equation (5) shows the procedure of computation.

(5) $$NPI=\sqrt[{2}]{\frac{{\left({CF}_{\max}\right)}^{2}+{\left({CF}_{mean}\;\right)}^{2}}{2}}$$

The metal pollution index (MPI) specifically assesses the contamination of water by heavy metals. It is determined by summing the ratio of the heavy metal concentration (C_i_) to the utmost permissible concentration for each heavy metal (C_max_).

(6) $$MPI=\sum_{i=1}^{i=n}\frac{C_{i}}{C_{\max}}$$

Water pollution can be classified as low to high based on these indices, displayed in Table 1.

### 2.4 Possible ecological risk analysis

In this study, possible ecological risk (PER) was evaluated by applying equations (7), (8)) [60]. ERI indicates the ecological risk of individuals, whilst TRF denotes the toxic response factor of individual metals. The TRF of Cd (30), Cu (5), Ni (5), Pb (5) [61]. The CI indicates the individual concentration which can be obtained by dividing measured concentration by the maximum allowable concentration of metals. Based on results, ecological risk can be identified as low (PER <150), medium (150 ≤ PER <300), considerable (300 ≤ PER <600) and extreme (PER ≥600) [55].

(7) $$ERI=TRI\times CI=TRI\times \frac{Mi}{Si}$$

(8) $$PER=\sum_{i=1}^{i=n}ERI$$

### 2.5 Health risk analysis

The health risk assessment of drinking water entails identifying contaminants that can adversely affect human health [60]. The intake of pollutants into the human body involves two major exposure pathways [57]. The first is the drinking water consumption or oral pathway, while the second is the dermal or skin contact pathway [62]. The United States Environmental Protection Agency initially developed this health risk assessment procedure [63]. This study assesses the health risks of oral intake and dermal contact for adults and children, separately. The noncarcinogenic risk for ingestion and dermal absorption is evaluated using equations (9), (10), (11), (12), (13)) [5,64]. Equation (13) computes total noncarcinogenic risk (HI) by summing the hazard score of ingestion and dermal contact.

(9) $${ADI}_{ing.}=\frac{C\times IR\times EF\times EP}{BW\times AT}$$

(10) $${HQ}_{ing.}=\frac{{CDI}_{ing.}}{{RfD}_{ing.}\;}$$

(11) $${ADI}_{der.}=\frac{C\times SA\times Kp\times ET\times EF\times EP\times CF}{BW\times AT}$$

(12) $${HQ}_{der.}=\frac{{ADI}_{der.}}{\;{RfD}_{der.}}$$

(13) $$HI=\sum_{i=1}^{n}{HQ}_{ing.}+{HQ}_{der.}$$

Here, ADI is the average daily intake (μg/kg-day) through ingestion (*ADI*_*ing*._) and dermal absorption (*ADI*_*der.*_), C is the measured metal concentration in water (μg/L) [5], IR denotes water ingestion rate (2.0 L/day for male, 1.6 L/day for female and 1.0 L/day for child), EF means exposure frequency (350 days/year), BW for body weight (70 kg for male, 55 kg for female and 16 kg for child), AT denotes average time (EP × 365 days), EP indicates exposure period (30 years for adults and 6 years for child), SA for exposed skin area (19000 cm^2^ for male, 16000 cm^2^ for female and 6800 cm^2^ for child) [20], Kp denotes coefficient of dermal permeability (0.001 cm/h for Cu and Cd, 0.004 cm/h for Pb [5], 0.0002 cm/h for Ni [22]), ET means exposure time (0.5 h/day for male, 0.75 h/day for female and 1.0 h/day for child), and CF indicates unit conversion factor (0.0001 L/cm^3^) [5]. The toxic reference dose of ingestion (RFD_ing_) for Pb = 1.4, Cd = 0.5, Cu = 40, Ni = 20 μg/kg-day, whilst the toxic reference dose of dermal absorption (RFD_der_) for Pb = 0.42, Cd = 0.005, Cu = 12, and Ni = 5.4 μg/kg-day [22,65].

The value of HQ < 1 are considered safe and non-carcinogenic, whereas HQ > 1 may represent a substantial noncarcinogenic risk [5,66]. The hazard index (HI) expresses the entire noncarcinogenic risk. A result of HI < 1 suggests the noncarcinogenic risk is acceptable, whereas HI > 1 indicates the risk exceeds the permissible limit [38].

Equations (14), (15), (16)) determine the carcinogenic risk (CR). Carcinogenic risk denotes the probability of a person developing any cancer over a lifetime from 24 h per day exposure to a carcinogenic factor for seventy years [67]. Here, ADI denotes average daily intake in mg/kg-day, and CSF indicates cancer slope factor (Cd = 6.1, Ni = 0.84, and pb = 8.5 kg/day/mg [22,67]. The acceptable thresholds are 10^−6^ for a single carcinogenic component and <10^−4^ for multi-element carcinogens [67].

Carcinogenic risk from ingestion,

(14) CRI=ADI(ing.)×CSF

Carcinogenic risk from dermal absorption

(15) CRD=ADI(der.)×CSF

Total carcinogenic risk,

(16) CR=CRI+CRD

### 2.6 Water quality analysis

The water quality index is the most effective approaches for evaluating suitability of water for drinking [68]. The current study implemented weighted arithmetic average to evaluate drinking water quality (Equations (17), (18), (19))). Equation (17) represents the quality rating of individual parameters. Here, V_m_ means measured value, Vs. for WHO standard values for drinking water parameters, and Vi indicates the ideal value of parameters (Vi = 0 for all parameters, except pH = 7) [22].

(17) $$Q_{i}=100*\;\left(\frac{V_{m}{-V}_{i}}{V_{s}{-V}_{i}}\right)$$

Equation (18) denotes the relative weight of individual parameters. It is typically inversely proportional to the standard value [68].

(18) $$W_{i}=\frac{1}{V_{s}}$$

Equation (19) can ascertain the ultimate water quality based on the quality rating and relative weight derived from preceding equations. Based on WQI, water can be categorized as: Excellent (WQI: 0–25), Good (WQI: 26–50), Poor (WQI: 51–75), Very poor (WQI: 76–100), and unsuitable for drinking (WQI>100) [69].

(19) $$WQI=\frac{\sum W_{i}Q_{i}}{\sum W_{i}}$$

### 2.7 Statistical analysis

The descriptive analysis was performed in this study to show the central tendency, dispersion, and shape of data set. It includes computation of minimum, maximum, arithmetic mean, standard deviation etc. This study employed version 20 of IBM's SPSS program for descriptive analysis, while Microsoft 365 had been used for additional analysis. Inferential statistics including correlation, principal component (PCA) and cluster (CA) were also employed in this study for determining temporal/spatial variations of water parameters [70,71]. The correlation analysis was applied to ascertain the associations and interrelationship among the different water parameters [3]. The PCA and CA were used to investigate possible sources and similar group of origin [61]. In this study the correlogram, PCA, heatmap was generated using software R (version: v.4.1.0). The library ‘ggplot2’ and ‘GGally’ for correlogram, ‘ggplot2’ and ‘factoextra’ for PCA [72] and ‘pheatmap’ for heatmap and hierarchical clusters (distance = Euclidean and method = ward.D2) [73] were adopted respectively. This study also incorporated spatial interpolation maps following Inverse distance weighting (IDW) method using software ArcGIS (version: 10.8.2).

## 3 Results and discussion

### 3.1 Descriptive analysis of water parameters around study area

Table 2 depicts the descriptive analysis of water parameters. The pH of collected samples varied from 7.24 to 8.2, with an arithmetic mean of 7.86 ± 0.26. Water is classified as acidic, basic, or neutral based on pH [70]. All samples are within the ranges of pH (6.5–8.5), as established by the WHO (2017), USEPA (2012), ISDW (2012), and BDWS (Table 2). It is ensuring 100 % compliance. According to recent studies, the mean value of pH surrounding the coastal region of Bangladesh observed as 7.2 in Khulna [74], 7.38 adjacent to Bay of Bengal [75], and 7.85 in the southeast coastal area [47]. A very low or higher pH level may increase the solubility of toxic metals in groundwater, which can directly affect human health through ingestion [47]. This outcome denotes that the analyzed water is safe for drinking and other general applications from the perspective of pH [76]. The value of alkalinity ranged from 361.9 to 515 mg/L, with an arithmetic mean of 452.1 ± 43.5 mg/L around study area. Although all samples conformed to the WHO guideline (200–600 mg/L), hardly 15 % adhered to the USEPA limit (30–400 mg/L), and none satisfied the Bangladeshi drinking water standards (20–200 mg/L). The water rich in alkalinity indicates the presence of substantial bicarbonates and carbonates. Although this cannot pose a direct health concern, it can lead to scaling and affect the taste of water [76]. Studied also found that the excessive alkalinity disrupts the body's normal pH by neutralizing and limiting the release of gastrointestinal fluids in humans [47].

Electrical conductivity indicates the overall concentration of dissolved constituents, including salt, organic, and inorganic substances in water. The amount of EC can influence on taste of water [77]. The arithmetic mean of EC concentration was observed as 1522 ± 946.8 μS/cm with a range of 770–4800 μS/cm in this study. Although 70 % of the samples met the USEPA and BDWS standards (50–1500 μS/cm), none conformed to the WHO threshold of 250 μS/cm (Table 2). This study revealed comparable results of significant saline content to previous studies conducted in southeast coastal regions of Bangladesh [40,47]. High conductivity indicates the presence of dissolved salts and minerals in the water, which may hint to potential pollution from salts or other substances [78]. Total dissolved solids (TDS), also indicates the presence of salt and organic matter in water [79]. The increased TDS levels may result from anthropogenic activity, reflecting ion concentrations and their mobility [77]. The study demonstrated TDS ranging from 450.6 to 3072 mg/L, with an arithmetic mean of 971.9 ± 607.8 mg/L. Approximately 75 % of samples conformed to the WHO and BDWS thresholds of 600–1000 mg/L and 1000 mg/L, respectively; however, hardly 5 % complied with the more stringent USEPA standard of 500 mg/L. The elevated TDS in numerous samples indicates a substantial presence of dissolved components, including salts or organic matter, which may affect the taste of water and quality [3].

Another water quality parameter, total hardness (TH) was found between 45 and 665 mg/L, with an arithmetic mean of 151.9 ± 128.1 mg/L. Most of the samples (90–95 %), complied with the WHO, USEPA, and BDWS standards, which range from 200 to 500 mg/L. The total hardness of water is attributable to calcium and magnesium ions derived from soil, rock, sediment, and minerals. Both low and excessive levels of hardness are detrimental to human health [47]. The water has high levels of calcium and magnesium, as indicated by its mean hardness value. Additionally, turbidity varied from 0.04 to 7.19 FTU, with an arithmetic mean of 1.17 ± 1.55 FTU. Between 95 % and 100 % of the samples were within the limits set by WHO, USEPA, and BDWS, which range from 4 to 10 FTU. Turbidity in water is caused by the presence of suspended, colloidal, and organic matter originating from soil or sediment, sludge, plankton, bacteria, and iron or aluminum hydroxide [87]. Increased turbidity may occur during rainfall, leading to digestive disorders, sedimentation issues, distortion, and health complications [87]. The water in this study generally clear and free from large amounts of suspended particles, making it suitable for consumption and meeting aesthetic quality standards. The arithmetic mean of free CO_2_ was obtained as 8.85 ± 2.86 mg/L with a range of 4–14 mg/L. The concentration of CO_2_ is generally below 10 mg/L in natural waters; lower CO_2_ levels are not concerning, whereas elevated CO_2_ might decrease pH, resulting in increased acidity and corrosiveness [88]. Approximately 75 % of the samples are within the limit of 10 mg/L; however, BDWS does not provide a precise limit, providing comparison with other unfeasible standards (Table 2). Most of the results fall within satisfactory limits; however, elevated levels in some cases may lead to problems such as corrosion in water pipes [3]. The oxidation-reduction potential (ORP) ranged from −64.3 to −21.5, with an arithmetic mean of −42.13 ± 10.62 throughout this investigation. ORP measures the water's ability to oxidize or reduce chemicals, which directly impacts biological activity and water quality [89]. A higher ORP with a positive score indicates better water quality. This study reveals a negative ORP, indicating lowering conditions that may signify poor water quality and possible organic pollution [89,90]. However, additional examinations using other criteria are required to assess overall water health.

The arithmetic mean of salinity obtained as 0.52 ± 0.50 ppt, with a range of 0.15–2.33 ppt. Increased salinity adversely affects water quality, enhances metal leaching, compromises water infrastructure, causing water hazardous for human consumption [3,91]. Ninety percent of the samples were below the USEPA limit of 1 ppt, while 75 % were under the BDWS limit of 0.66 ppt. The elevated amount of salinity may originate from different levels of chloride (NaCl, KCl, CaCl_2_ and so on) [40,47,74]. This study identified as NaCl (140.3–2127.2 mg/L, with an arithmetic mean of 472.6 ± 459.7 mg/L) and Cl^−^ (85.1–1290.4 mg/L, with an arithmetic mean of 472.6 ± 278.6 mg/L). NaCl concentrations of 40 %–75 % of samples were beyond acceptable limits, while Chloride concentrations exceeded the suggested thresholds in 85 % of samples. The previous investigation in southeast coastal part of Bangladesh also found similar results [40,47,74]. The higher concentration of salinity in this part may result from sea water instruction, brine shrimp aquaculture, and so on [40,47,74]. In this investigation the arithmetic mean of Ca^2+^ obtained as 60.78 ± 50.88 mg/L with range of 18.04–264.5 mg/L. Calcium is a naturally occurring mineral in water, primarily sourced from the dissolution of rocks. Calcium levels in the water samples are mostly within allowable limits, posing no substantial health risks [76].

This investigation examined heavy metals including Cd, Cu, Ni, and Pb. The arithmetic mean of these metals were determined to be 0.026 ± 0.038 mg/L, 0.008 ± 0.005 mg/L, 0.084 ± 0.089 mg/L, and 0.213 ± 0.055 mg/L, respectively. The samples' 70 %, 55 %, 100 %, and 65 % meet the WHO criteria for Cd, Cu, Ni, and Pb, respectively. Meanwhile, 95 %, 55 %, 100 %, and 70 % are within the acceptable range of BDWS (Table 2). The number of heavy metals in water is a global issue due to its detrimental impact on living organisms. Cadmium is regarded as harmful due to its prolonged half-life [92]. It may originate from natural or anthropogenic sources; can accumulate in aquatic organisms and plants, consequently entering the food chain. The consumption of excessive Cd can result in acute poisoning, leading to diarrhea and impairing renal and hepatic function [87]. Elevated levels of copper (Cu) in drinking water can pose several health hazards [93]. Acute exposure to high levels may lead to gastrointestinal disorders, such as nausea, vomiting, and diarrhea. Long-term exposure may cause hepatic and renal impairment, potentially leading to hepatic and renal failure [94]. Nickel and lead are carcinogenic metals. The excessive nickel in conjunction with other metals can harm DNA; it is also associated with conjunctivitis, asthma, and inflammatory responses [87,92]. The elevated amount of Pb is a matter of concern for developing nations. Lead exposure can result in neurological disorders, renal impairment, anemia, arthritis, dyslexia, autism, and various congenital abnormalities [95].

**Fig. 2:**
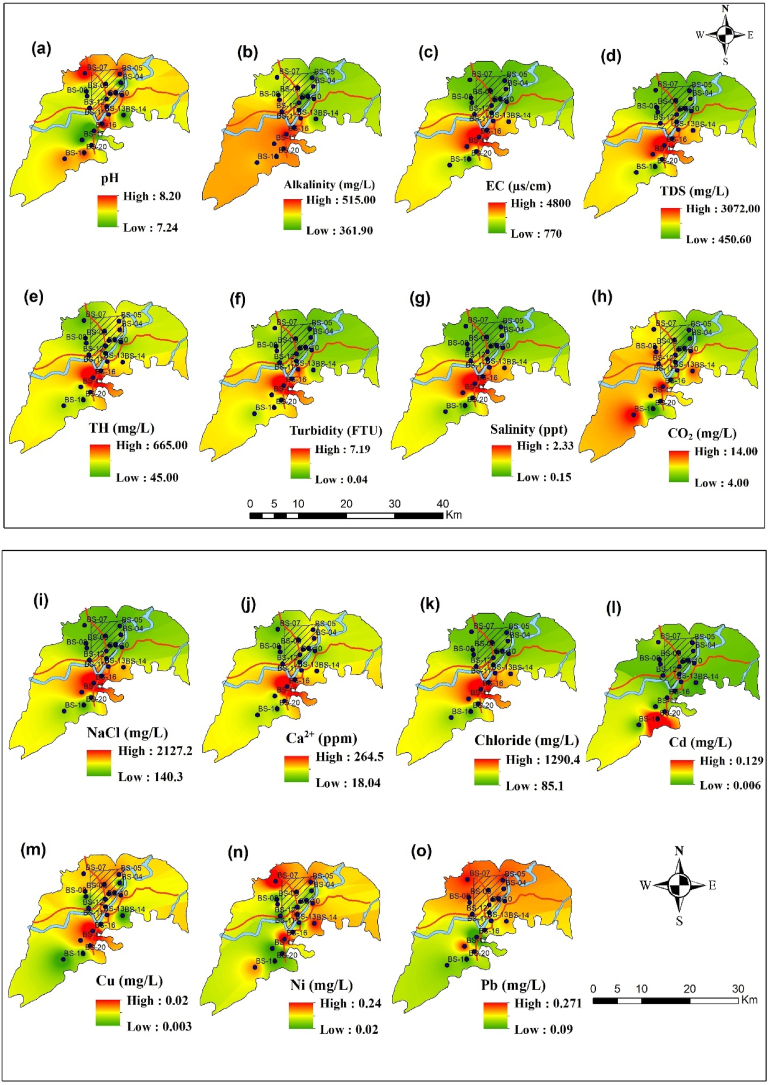
Spatial interpolation shows the distribution of water parameters around study area. Here, (a) indicates the distribution of pH, (b) for alkalinity, (c) for electrical conductivity (EC), (d) for total dissolved solids (TDS), (e) denotes total hardness (TH), (f) for turbidity, (g) for salinity, (h) for free CO_2_, (i) NaCl, (j) for Ca^2+^, (k) for chloride, (l) for cadmium, (m) copper, (n) for nickel and (o) lead.

Fig. 2 illustrates the spatial interpolation of the analyzed water parameters. The spatial distribution of water quality parameters highlights distinct variations across the study area, offering insights into pollution levels and potential contamination sources. The intensity of red, yellow, and green colors signifies high, medium, and low concentrations of each parameter, respectively. The pH distribution in Fig. 2 (a) exhibits homogenous and medium concentrations except for the central part, where the concentration is low. In contrast, Alkalinity displayed in Fig. 2(b) shows increased concentrations toward the central and southern zones, potentially influenced by agricultural or industrial runoff.

However, EC (c) TDS (d), Turbidity (f), Salinity (g), and NaCl (i) exhibit similar pattern distribution of their concentration (Fig. 2). They show the low concentration in the north-eastern part, medium concentration in southern part but the high concentration in the eastern central part of the study area. These high concentration values point to dissolved ions, often linked to pollutants such as heavy metals, saline intrusion or chemical contamination from industries, thus implying anthropogenic influence in this region. In addition, particulate matter accumulation could be attributed to sediment transport or pollution runoff, as turbidity is typically linked to disturbances or contamination in the water source. Furthermore, TH (e) and CO₂ (h) follows a similar pattern, reinforcing the idea that dissolved ions, particularly those contributing to water hardness, are concentrated in the southern zones. These high concentrations are likely linked to industrial activities including anthropogenic activities that are a key contributor to water quality degradation in this region. However, different specific ions and metals, Ca^2^⁺ (j) and chloride (k) show high concentrations in the southern region, particularly around BS-16 and BS-18. The Cd (l), Cu (m), Ni (n), and Pb (o) also exhibit higher concentrations in central to southern parts of the study area, particularly around BS-16 and BS-18. These heavy metals, especially Cd and Pb, present significant environmental hazards due to their toxicity and potential for bioaccumulation, thus posing risks to both ecosystems and human populations.

### 3.2 Contamination scenario of water around study area

Numerous researchers around the globe developed multiple indices for assessing the pollution scenario of metal presence in water [3]. This study incorporated all water parameters along with metals to attain the final outcomes. All contamination evaluated regarding the background value (Si) complied by the environmental conservation regulations of Bangladesh [82].Table 3 represents the results.

**Table 3:** Assessment of contamination scenarios in the investigated region employing multiple pollution indices.

Sample ID	Total contamination Index			Modified contamination index			Pollution Load Index			Nemerow Pollution Index			Metal pollution index			Weighted contamination index	
	TCI	Remarks	AW	MCI	Remarks	AW	PLI	Remarks	AW	NPI	Remarks	AW	MPI	Remarks	AW	WCI	Remarks
BS-1	17.72	high	4	1.18	very low	1	0.49	low	2	3.43	medium	3	2.21	medium	3	0.19	low
BS-2	7.08	low	2	0.47	very low	1	0.50	low	2	1.32	low	2	0.00	very low	0	0.12	low
BS-3	7.38	low	2	0.49	very low	1	0.46	low	2	1.51	low	2	0.00	very low	0	0.12	low
BS-4	9.38	medium	3	0.63	very low	1	0.63	medium	3	1.66	low	2	0.00	very low	0	0.15	low
BS-5	17.12	high	4	1.14	very low	1	0.48	low	2	3.90	medium	3	1.91	medium	3	0.17	low
BS-6	7.51	low	2	0.50	very low	1	0.47	low	2	1.75	low	2	0.00	very low	0	0.12	low
BS-7	19.63	high	4	1.31	very low	1	0.59	medium	3	3.94	medium	3	2.15	medium	3	0.19	low
BS-8	7.88	low	2	0.53	very low	1	0.51	medium	3	1.81	low	2	0.00	very low	0	0.13	low
BS-9	8.31	medium	3	0.55	very low	1	0.56	medium	3	1.52	low	2	0.00	very low	0	0.15	low
BS-10	8.34	medium	3	0.56	very low	1	0.44	low	2	1.43	low	2	0.00	very low	0	0.13	low
BS-11	16.99	high	4	1.13	very low	1	0.56	medium	3	3.17	medium	3	2.02	medium	3	0.19	low
BS-12	10.22	medium	3	0.68	very low	1	0.66	medium	3	1.80	low	2	0.00	very low	0	0.15	low
BS-13	20.17	high	4	1.34	very low	1	0.98	medium	3	3.20	medium	3	1.65	medium	3	0.19	low
BS-14	12.51	medium	3	0.83	very low	1	0.89	medium	3	1.58	low	2	0.00	very low	0	0.15	low
BS-15	21.30	high	4	1.42	very low	1	0.80	medium	3	3.54	medium	3	1.46	medium	3	0.19	low
BS-16	36.57	very high	5	2.44	medium	3	1.67	high	4	4.04	medium	3	2.51	high	4	0.25	medium
BS-17	14.45	medium	3	0.96	very low	1	0.99	medium	3	1.86	low	2	0.00	very low	0	0.15	low
BS-18	17.00	high	4	1.13	very low	1	0.69	medium	3	2.91	medium	3	1.78	medium	3	0.19	low
BS-19	9.29	medium	3	0.62	very low	1	0.63	medium	3	1.82	low	2	0.00	very low	0	0.15	low
BS-20	36.49	very high	5	2.43	medium	3	1.62	high	4	3.98	medium	3	2.45	medium	3	0.23	medium

The total Contamination Index (TCI) is a cumulative measure of contamination across various pollutants. It evaluates the total contamination level by summing the concentrations of different pollutants in a particular area. It categorized water contamination from low to very high levels [55]. Sample IDs such as BS-2, BS-3, and BS-6 exhibit minimal contamination, with TCI values ranging from 7.08 to 7.88. Mild contamination is observed in BS-4, BS-9, and BS-10 samples. The samples including BS-1, BS-5, BS-7, and BS-18 exhibit elevated contamination levels. The most alarming samples are BS-16 and BS-20, with TCI values exceeding 36.

The Modified Contamination Index (MCI) enhances the assessment of contamination by using designated weights. It evaluates the severity of each pollutant, offering a comprehensive analysis [56]. The study reveals that most samples, specifically BS-2, BS-3, and BS-6, demonstrate minimal contamination, while samples BS-13, BS-14, and BS-17 show moderate contamination. The samples BS-16 and BS-20 demonstrate increased overall contamination. In addition, the Pollution Load Index (PLI) is a holistic metric that evaluates the aggregate contamination intensity from many sources and pollutants [96]. This index also categorized contamination into low, medium, and high [45]. In this study, BS16 and BS 20 exhibit an elevated PLI, whereas the pollution levels of the remaining samples range from moderate to low. Moreover, the Nemerow Pollution Index (NPI) is an advanced tool for evaluating environmental hazards using many pollution characteristics. In contrast to other indices, the NPI prioritizes the entire risk presented by pollutants, considering their capacity to affect the ecology and the environment adversely [22]. NPI indicates medium to low levels of pollution for all samples. The MPI used in the present study for measuring pollution from heavy metals. The samples BS-16 exhibits high metal pollution, suggesting significant environmental risks associated with toxic heavy metals. Based on MPI, the samples including BS 1, BS-7, BS-11, BS-18, and BS 20 indicate moderate pollution, representing mild concentration of trace metal toxicity. The remaining samples have a lower level of pollution.

The weighted contamination index (WCI) is a novel approach for assessing pollution. It is obtained by integrating all existing contamination indices and combining numerous metrics into a single platform to achieve the results [3]. WCI offers a more precise representation of a data set than the normal arithmetic mean. According to WCI, BS 16 and BS 20 represent moderate levels of contamination, and the rest of the samples have lower levels of contamination.

**Fig. 3:**
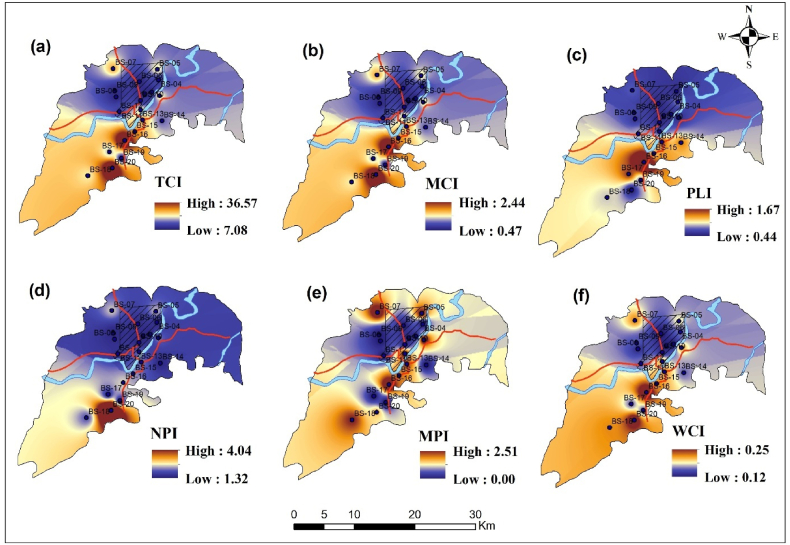
Spatial map representing contamination scenario around study area. In this figure (a) represents spatial interpolation of total contamination index (TCI), (b) indicates modified contamination index (MCI), (c) for pollution load index (PLI), (d) denotes Nemerow pollution index (NPI), (e) for metal pollution index (MPI), and (f) indicates weighted contamination index (WCI).

The samples including BS-2, BS-3, and BS-6 consistently demonstrate lower pollution levels across all metrics. Moreover, BS-9, BS-10, and BS-14 demonstrate moderate level of pollution according to TCI and PLI values. Although these places are not significantly contaminated, they signify prospective danger zones where pollution could intensify without appropriate intervention. BS-1, BS-5, and BS-7 reflect significant contamination across multiple indices, underscoring areas of concern that may necessitate targeted environmental management and pollution mitigation strategies. BS-16 and BS-20 exhibit the highest degrees of contamination and pollution, characterized by elevated TCI and NPI values. The samples indicate pressing environmental hazards, especially heavy metal contamination, requiring prompt remediation. The spatial interpolation of contamination indices displayed in Fig. 3. The pollution indices provide a cumulative measure of contamination across various parameters, highlighting areas under significant stress. The TCI (a) marks the southern region near BS-20 as the most heavily contaminated indicating severe pollution levels. The MCI (b) shows a similar pattern, albeit with more moderate values indicating collective contamination from multiple pollutants. Furthermore, the PLI (c) and NPI (d) point to significant stress in the southern parts of the study area indicating these regions are heavily impacted by contamination. Additionally, the MPI (e) also reaches its peak in the southern zones, confirming earlier findings of concentrated pollution. The WCI (f) although lower, still indicates moderate pollution levels, with the southern regions once again standing out as areas of concern. These indices collectively underscore that the southern part of the study area experiences the highest contamination, likely driven by industrial effluents or untreated wastewater discharges.

### 3.3 Possible ecological risk of water around study area

**Fig. 4:**
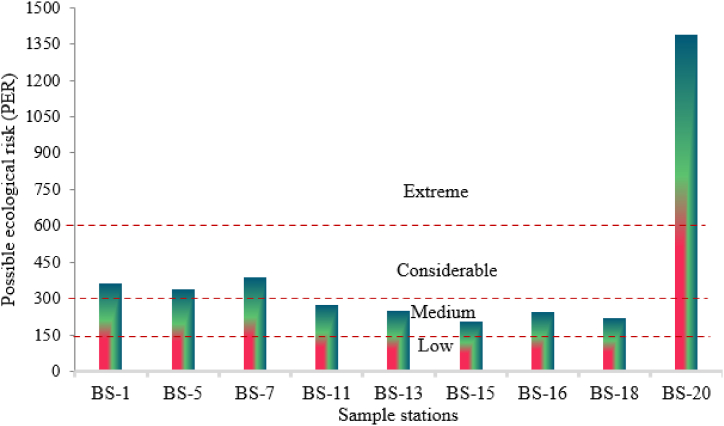
Possible ecological risk of metals presence in water samples.

Ecological risk is a useful tool for evaluating the potential detrimental effects of metals on ecosystems and the environment. The ecological risk of individual metals (ERI) is initially evaluated to determine the potential ecological risk (PER). Based on ERI risk of individual metals can be classified as low: ERF <40, moderate: 40 ≤ ERF <80, considerable: 80 ≤ ERF <160, high: 160 ≤ ERF <320, and very high: ERF ≥320 [97]. Table 4 illustrates the ecological danger associated with metals, while Fig. 4 depicts the overall threat. The ERI for Cd ranges from 60 to 1290 with an arithmetic mean of 264.4. The arithmetic mean of ERI for Cd was identified to be significant (160 ≤ ERF <320), while for BS20, it was classified as exceedingly high. The individual risk of copper was found to be low (<40) in all instances, while the mean concentration of nickel was also minimal throughout the area of concern. The ecological risk of Pb varies from 45 to 135.5 with an arithmetic mean of 106.7, falling within considerable (: 80 ≤ ERF <160, high) ranges [98].

**Table 4:** Ecological risk of individual metals presence in water samples.

Sample ID	Ecological Risk Indicator (ERI)			
	Cd	Cu	Ni	Pb
BS-1	200	0.8	42.5	117.5
BS-5	160	0.9	42.5	135
BS-7	190	0.7	60	135.5
BS-11	110	0.8	55	108.5
BS-13	140	0.8	2.5	108
BS-15	60	0.4	25	120
BS-16	140	2	60	45
BS-18	90	0.1	32.5	99
BS-20	1290	0.3	5	92
Minimum	60	0.1	2.5	45
Maximum	1290	2	60	135.5
Arithmetic mean	264.4	0.76	36.1	106.7

The total potential ecological risk (PER) varies from 205.4 to 1387.3, with an arithmetic mean of 408.2 (Fig. 4). The PER of BS 20 is extremely high (>600), whereas BS1, BS5, and BS7 are within a reasonable range. The remaining samples demonstrate a moderate level of ecological risk [61]. The peak value of PER at BS 20 was attributable to elevated concentrations of Cd and Pb detected in that location. In conclusion, the ecological risk assessment reveals that Cd is the most significant hazard, exhibiting considerable variability and a heightened average risk, followed by Pb, which demonstrates moderate risk in certain regions. Nickel exhibits moderate risk with reduced variability, whereas copper demonstrates the least ecological danger. For regions with substantial cadmium and lead contamination, early action is recommended; however, nickel and copper require less immediate attention [99,100].

### 3.4 Possible health risk of studied water samples

The health risk (HR) assessment established by the USEPA is an essential tool for decision-makers to understand the potential ramifications of actions, treatments, disposal, mitigation, and water cleanup standards [20]. This study incorporated noncarcinogenic risk (NCR) and carcinogenic risk (CR) assessments to estimate the potential health effects of heavy metals in water. The noncarcinogenic risk includes the hazard score of ingestion and dermal absorption of drinking water. Table 5 represents the results, where NCR was evaluated for children, and adult (male/female) individually. The hazard index of ingestion ranges from 3.93E-03 to 1.86E-02, dermal absorption from 3.21E-06 to 5.27E-05, and total noncarcinogenic risk from 3.94E-03 to 1.86E-02 for children. For adults (male), HI of ingestion varies from 1.80E-03 to 1.02E-04, with an arithmetic mean of 3.10E-03; HI dermal absorption ranges 1.11E-05 to 1.02E-04. And total noncarcinogenic risk was obtained between 1.82E-03 and 8.60E-03, with an arithmetic mean of 3.13E-03. Moreover, for adults (female), HI ingestion was found within 1.83E-03 and 8.65E-03; HI (dermal) was observed between 1.26E-05 to 1.17E-04, with total NCR from 1.86E-03 to 8.77E-03. The hazards score for noncarcinogenic risk was obtained within acceptable limits (<1) throughout this study [67]. However, the findings indicate that the HI and NCR for children surpass those of adults, and these values for females exceed those of males [101]. It suggests that youngsters are more susceptible to ingestion-based exposure than adults, with girls being more vulnerable than males [21,22]. Excess non-cancer elements in water can harm the liver, skin, causing nausea, vomiting, diarrhea, abdominal pain, sickness, dizziness, asthma, allergic reactions, heart disorders, immune imbalances, anemia, hypertension, gastrointestinal effects, skeletal delay, infertility, and so on [102]. The combined hazard score of less than one indicates no potential non-carcinogenic risk connected with the consumption of water around the studied region [103].

**Table 5:** The noncarcinogenic risk assessment for children, male and female around study area.

Sample ID	Children			Adult (male)			Adult (female)		
	HI (I∗)	HI (D∗)	NCR∗	HI (I∗)	HI (D∗)	NCR∗	HI (I∗)	HI (D∗)	NCR∗
BS-1	6.83E-03	9.46E-06	6.84E-03	3.12E-03	2.76E-05	3.15E-03	3.18E-03	3.16E-05	3.21E-03
BS-5	6.94E-03	7.83E-06	6.94E-03	3.17E-03	2.45E-05	3.20E-03	3.23E-03	2.80E-05	3.26E-03
BS-7	7.52E-03	9.55E-06	7.53E-03	3.44E-03	3.17E-05	3.47E-03	3.50E-03	3.62E-05	3.54E-03
BS-11	5.60E-03	6.15E-06	5.61E-03	2.56E-03	2.41E-05	2.59E-03	2.61E-03	2.75E-05	2.64E-03
BS-13	5.29E-03	5.77E-06	5.29E-03	2.42E-03	1.11E-05	2.43E-03	2.46E-03	1.26E-05	2.47E-03
BS-15	5.02E-03	3.21E-06	5.02E-03	2.30E-03	1.17E-05	2.31E-03	2.34E-03	1.34E-05	2.35E-03
BS-16	3.93E-03	7.62E-06	3.94E-03	1.80E-03	2.80E-05	1.82E-03	1.83E-03	3.20E-05	1.86E-03
BS-18	4.76E-03	4.61E-06	4.77E-03	2.18E-03	1.61E-05	2.19E-03	2.22E-03	1.84E-05	2.24E-03
BS-20	1.86E-02	5.27E-05	1.86E-02	8.50E-03	1.02E-04	8.60E-03	8.65E-03	1.17E-04	8.77E-03

HI= Hazard index, I∗ = Ingestion, D∗ = Dermal absorption, NCR= Non carcinogenic risk.

**Fig. 5:**
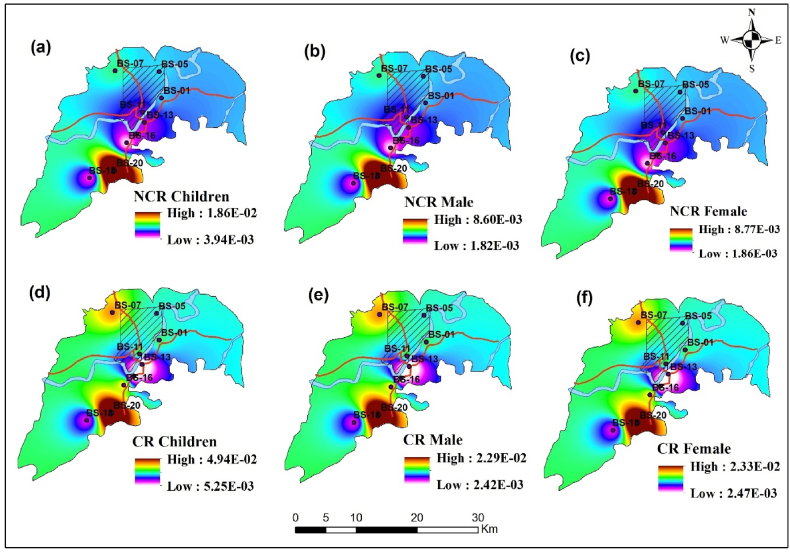
Spatial distribution of health risk around the study area. The upper series indicates noncarcinogenic risk (NCR) and lower series represents carcinogenic (CR) for children, adult (male) and adult (female), respectively.

However, the carcinogenic risk of heavy metals (Cd, Ni, and Pb), evaluated in this study is presented in Table 6 and Fig. 5. For children, the CR for Cd varied from 2.20E-03 to 4.73E-02, with an arithmetic mean of 1.05E-02, indicating significant variability, particularly due to the high value in BS-20. Nickel ranges from 2.04E-03 to 2.45E-02, with an arithmetic mean of 1.42E-02. However, Pb has consistently minimal risk, ranging from 4.60E-05 to 1.39E-04, with an arithmetic mean of 1.13E-04 and standard deviation of 3.06E-05. The CR of Cd for adults (male) ranged between 1.02E-03 to 2.19E-02, with an arithmetic mean of 4.86E-03, where CR for Ni varied from 9.45E-04 to 1.13E-02, with an arithmetic mean of 6.52E-03, And CR for Pb ranges from 2.13E-05 and 6.40 E−05 with an arithmetic mean of 5.35E-05. Moreover, the CR of Cd for females was obtained between 1.04E-03 to 2.23E-02, with an arithmetic mean of 4.96E-03; CR for Ni found between 9.64E-04 to 1.16E-02, and Pb from 2.17E-05 to 6.53E-05 with an arithmetic mean of 5.46E-05. For all demographic groups, Cd exhibits the highest variability in cancer risk, with high standard deviations due to elevated values in BS-20. Additionally, the outcomes also indicated that the carcinogenic risk of Cd and Ni was more significant than the international safe limit (1E-04) [57]. The elevated amount of CR from Ni may cause lung and nasal cancer, congenital heart defects, kidney damage, increase skin problems, and so on [101,104]. In addition, the risk of increasing amount of Cd directly linked to lung, jugular, pancreatic, prostate, and breast cancer, lowering the intelligence quotient, ingenuity, and causing detrimental health effects [20,101]. Moreover, the higher level of Pb may lead to neurological difficulties, kidney malfunction, anemia, renal dysfunction, arthritis, dyslexia, and so on [95,105]. However, children are more vulnerable to carcinogenic and noncarcinogenic risk than adults according to study, which matches previous studies [67,92].

**Table 6:** Carcinogenic risk (CR) of Cd, Ni, Pb for children and adults (male-female) around study area.

Sample ID	CR for children				CR for adult (male)				CR for adult (female)			
	Cd	Ni	Pb	CR	Cd	Ni	Pb	CR	Cd	Ni	Pb	CR
BS-1	7.34E-03	1.73E-02	1.20E-04	2.48E-02	3.39E-03	8.03E-03	5.55E-05	1.15E-02	3.46E-03	8.19E-03	5.66E-05	1.17E-02
BS-5	5.87E-03	1.73E-02	1.38E-04	2.33E-02	2.71E-03	8.03E-03	6.38E-05	1.08E-02	2.77E-03	8.19E-03	6.50E-05	1.10E-02
BS-7	6.97E-03	2.45E-02	1.39E-04	3.16E-02	3.22E-03	1.13E-02	6.40E-05	1.46E-02	3.28E-03	1.16E-02	6.53E-05	1.49E-02
BS-11	4.04E-03	2.24E-02	1.11E-04	2.66E-02	1.86E-03	1.04E-02	5.13E-05	1.23E-02	1.90E-03	1.06E-02	5.23E-05	1.26E-02
BS-13	5.14E-03	–	1.10E-04	5.25E-03	2.37E-03	–	5.10E-05	2.42E-03	2.42E-03	–	5.20E-05	2.47E-03
BS-15	2.20E-03	1.02E-02	1.23E-04	1.25E-02	1.02E-03	4.72E-03	5.67E-05	5.80E-03	1.04E-03	4.82E-03	5.78E-05	5.92E-03
BS-16	5.14E-03	2.45E-02	4.60E-05	2.97E-02	2.37E-03	1.13E-02	2.13E-05	1.37E-02	2.42E-03	1.16E-02	2.17E-05	1.40E-02
BS-18	3.30E-03	1.33E-02	1.01E-04	1.67E-02	1.53E-03	6.14E-03	4.68E-05	7.72E-03	1.56E-03	6.26E-03	4.77E-05	7.87E-03
BS-20	4.73E-02	2.04E-03	9.41E-05	4.94E-02	2.19E-02	9.45E-04	4.35E-05	2.29E-02	2.23E-02	9.64E-04	4.43E-05	2.33E-02
Minimum	2.20E-03	2.04E-03	4.60E-05	5.25E-03	1.02E-03	9.45E-04	2.13E-05	2.42E-03	1.04E-03	9.64E-04	2.17E-05	2.47E-03
Maximum	4.73E-02	2.45E-02	1.39E-04	4.94E-02	2.19E-02	1.13E-02	6.40E-05	2.29E-02	2.23E-02	1.16E-02	6.53E-05	2.33E-02
Mean	9.70E-03	1.64E-02	1.09E-04	2.44E-02	4.49E-03	7.61E-03	5.04E-05	1.13E-02	4.57E-03	7.78E-03	5.14E-05	1.15E-02

The spatial distribution of NCR and CR around Barishal sadar presented in Fig. 5(a–f). For NCR, children Fig. 5(a) are particularly vulnerable, with the highest risks concentrated around BS-18 and BS-20. This highlights the disproportionate impact of contaminants on children, who are more susceptible to the harmful effects of pollutants, including developmental and neurological disorders. Similar patterns are observed for both males Fig. 5 (b) and females Fig. 5 (c), though their NCR values are slightly lower than children. Similarly, children are also facing the greatest risk in case of carcinogenic risk Fig. 5 (d). This highest risk indicates potential long-term health impacts, such as cancer development, due to prolonged exposure to carcinogenic substances like cadmium and lead. The risks for both males Fig. 5 (e) and females Fig. 5 (f) are similarly concentrated in the southern regions [67,92]. These overlapping high-risk zones for both NCR and CR emphasize the critical need for pollution control measures to reduce health risks in these heavily affected regions.

### 3.5 Overall water quality around Barishal Sadar

The water quality index (WQI) is a commonly used measure for monitoring surface-groundwater pollution and implementing water quality improvement projects around the world [22]. This study used the weighted arithmetic method to get the outcomes, presented in Table 7. All examined water data, including physicochemical properties and metal concentrations, has been used in this calculation. The WQI ranged from 0.32 to 2247.20, with an arithmetic mean of 344.82 throughout this investigation. There have been four categories of water found within the study region according to the results. According to classification, 55 % of samples are categorized as excellent, with WQI scores between 0.32 and 0.64. In addition, 10 % of the samples are classified as moderate quality. Moreover, 15 % of samples fall below the poor range (200<WQI<300), whereas 20 % of samples are classified as very poor (WQI>300) [38,68]. The water quality index suggests that water in poor and extremely poor condition necessitates purification prior to consumption [22,45].The water samples classified as grade-1 excluded heavy metals concentrations, however those identified as class 3, 4, and 5 included these quantities. These outcomes also elucidate the relationship between heavy metals and water quality degradation around the investigated area [22].

**Table 7:** Water quality index and drinking water conditions around the area of concern.

**Samples**	**WQI**	**Water** **Grade**	**Remarks**	**Samples**	**WQI**	**Water** **Grade**	**Remarks**	**Dominancy (%)**
BS-1	391.18	5	Very poor	BS-11	235.89	4	Poor	**Excellent = 55 %**
BS-2	0.43	1	Excellent	BS-12	0.48	1	Excellent	
BS-3	0.61	1	Excellent	BS-13	277.99	4	Poor	**Good = 0 %**
BS-4	0.64	1	Excellent	BS-14	0.59	1	Excellent	
BS-5	328.81	5	Very poor	BS-15	149.19	3	Moderate	**Moderate = 10 %**
BS-6	0.40	1	Excellent	BS-16	268.16	4	Poor	
BS-7	383.26	5	Very poor	BS-17	0.50	1	Excellent	**Poor = 15 %**
BS-8	0.33	1	Excellent	BS-18	194.51	3	Moderate	
BS-9	0.38	1	Excellent	BS-19	0.51	1	Excellent	**Very poor = 20 %**
BS-10	0.32	1	Excellent	BS-20	2247.20	5	Very poor	

**Fig. 6:**
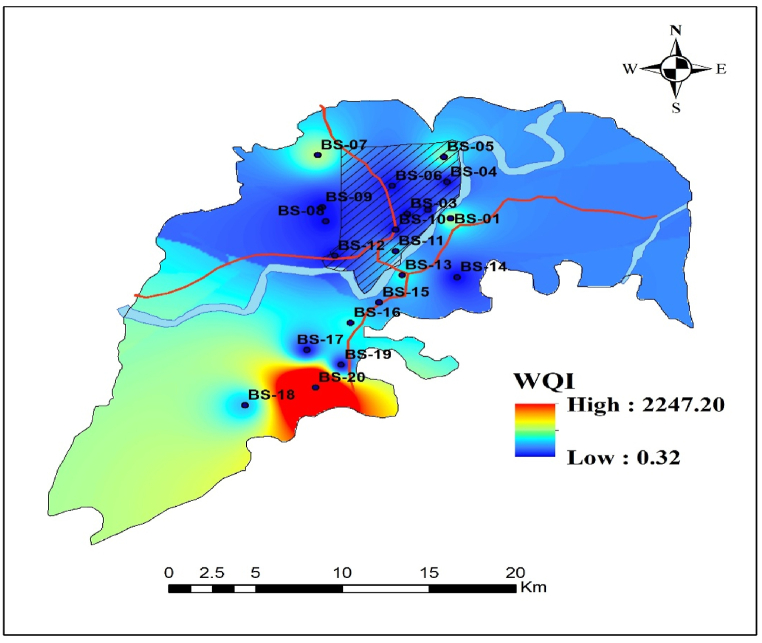
Spatial distribution of water quality index (WQI) around study area.

The spatial distribution of drinking water quality around Barishal Sadar is displayed in Fig. 6. The red color indicates higher value, where the blue color indicates the lower. The northern and central regions show low values of WQI that imply good water quality. That indicates good water practices for these cities and fewer industrial activities. In contrast, high WQI values are displayed over the southern part and more highlighting concern is the east-southern part (BS- 17, 17, 20) indicating very poor quality of water. These low values are likely due to agricultural runoff and industrial discharges that are major contributors to water quality degradation [106]. Moreover, the other areas show a moderate quality of water, indicating transitional zones influenced by both natural and anthropogenic activities.

However, these variations in water quality within the study area are not merely a reflection of the worse environmental condition but also demonstrate the pressing need for effective management of water quality for specific region priority. However, certain pollution indicators, such as total nitrogen, chemical oxygen demand and other heavy metals, play critical roles in determining overall water quality [107]. Furthermore, understanding the seasonal variability or dynamics is also an important factor because different pollutants show dominancy in different seasons (dry and wet seasons) [108]. Therefore, understanding seasonal variability provides deep insight into informing targeted interventions aimed at mitigating agricultural runoff and industrial discharge and managing other point sources effectively. This intricate approach is very urgent and essential for developing sustainable management strategies that safeguard both water quality and ecological health within diverse aquatic systems. Additionally, the local community's engagement in monitoring efforts can enhance the development of proper planning and management strategies including fostering a sense of stewardship which ultimately leads to more resilient water management practices.

### 3.6 Source apportionment of water parameters around investigation area

**Fig. 7:**
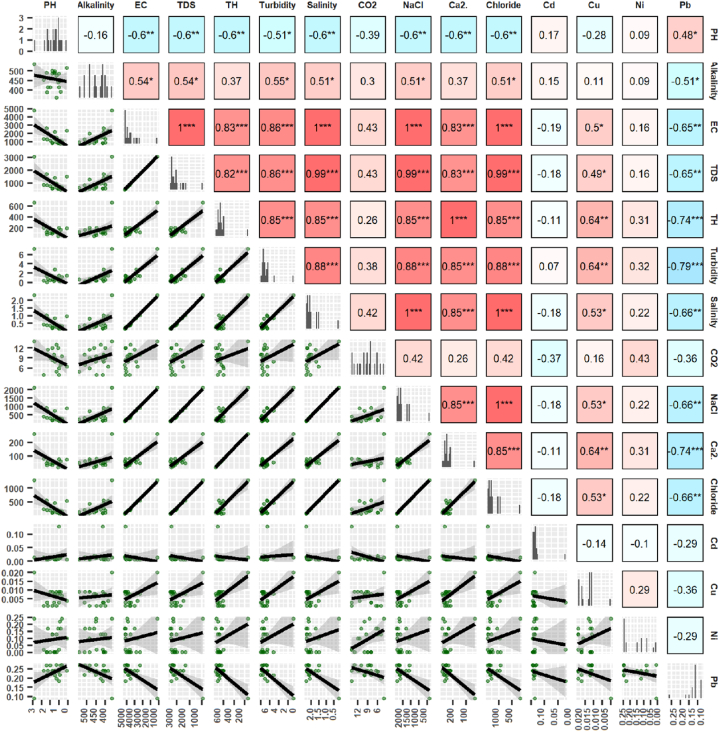
Correlogram indicating Pearson correlation and scatterplot of water parameters. Moreover, this study applied principal component analysis to determine a more specific identification of the source apportionment of water parameters around investigation area [112]. Fig. 8 represents the results. The first principal component (PC1), accounting for 65.55 % of the variation, is predominantly influenced by variables such as TDS, EC, NaCl, Chloride, and Salinity, signifying that dissolved salts and ionic content are the primary influencers of water quality. These factors are probably associated with natural mineral dissolution or agricultural runoff, indicating that elevated salinity levels are a significant feature of certain water samples, especially those in Group 1. These relations indicate the origin of these water parameters has the same pathways, and it may be the effect of slight saltwater intrusion though Barishal city locates closed to the Bay of Bengal [113].

The interrelationship, association and source apportionment of water parameter was determined using correlation matrix, principal component analysis and heat map clustering in this study. Correlation analysis is crucial for understanding the complex interaction among different water quality parameters. This method is useful to evaluate the association among distinct variables, may indicate pinpoint key factors influencing water quality. A correlogram is presented in Fig. 7. The upper triangular section of figure represents Pearson correlation coefficient, while the lower triangular represents the scatterplot of comparison between two variables. The correlation coefficient 1 indicates a positive perfect correlation, −1 denotes a perfect negative interrelation, and 0 for no correlation [70]. The positively significant relationship among variables indicated by reddish color, where negatively significant are bluish in Fig. 7. This study identified a robust significant correlation between EC and TDS (1.00∗∗∗, three asterisks imply p < 0.001), suggesting an almost linear relationship between both variables, as demonstrated by the scatterplot. EC has also a strong association with TH, turbidity, salinity, NaCl, and chloride. According to previous studies significant relations indicate the same sources, and insignificant relationships indicate different pathways [6,19].

**Fig. 8:**
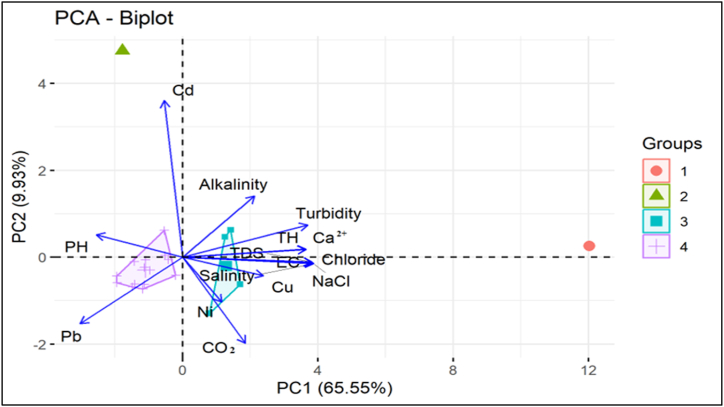
Diagram indicating principal component analysis of water parameters.

Moreover, the variables EC, TDS, salinity, turbidity, NaCl, Ca2+, and chloride have significant interrelation. This results indicated that these variables may originate from similar sources [109]. These sources may include higher amounts of ion from sodium, calcium or chloride salts. These may be associated with mineral dissolution, agricultural runoff, weathering of rocks, or industrial operations [3,110]. Since this investigation was undertaken in the southeast coastal region of Bangladesh, the increased level of salinity along with significant water parameters may be caused by saltwater instruction in the groundwater table surrounding Barishal Sadar [40,68]. The pH showed a weak to moderate correlation with most of the water parameters (TDS, TH, turbidity). It may indicate the acidic or alkaline conditions which can alter the solubility of different metals as well as minerals. Heavy metals in this study exhibit generally minor association with the other parameters, indicating their source of origin may be affected by more independent processes [111].

The PC2 represents 9.93 % of the variance. It explains the significant relation among turbidity, TH, and Calcium (Ca^2^⁺). On the other hand, parameters including alkalinity and pH substantially influence PC2. This result signifies an alternative aspect of water quality associated with acidity and buffering capacity, independent of salinity. The analyzed metals explain a modest impact on the overall variance; however, Group 2 appears more affected by Cd. This result represents the risk of metal pollution from localized industrial operations. Grouping patterns show that Group 1 is significantly affected by salinity and dissolved solids, whereas Group 2 exhibits a more pronounced correlation with metal contamination. Groups 3 and 4 demonstrate more moderate water quality attributes, featuring fewer extreme values for the variables. Overall, the PCA analysis emphasizes the primary elements affecting water quality and provides insights into probable contamination sources, which guide water management activities and pollution control measures [114].

**Fig. 9:**
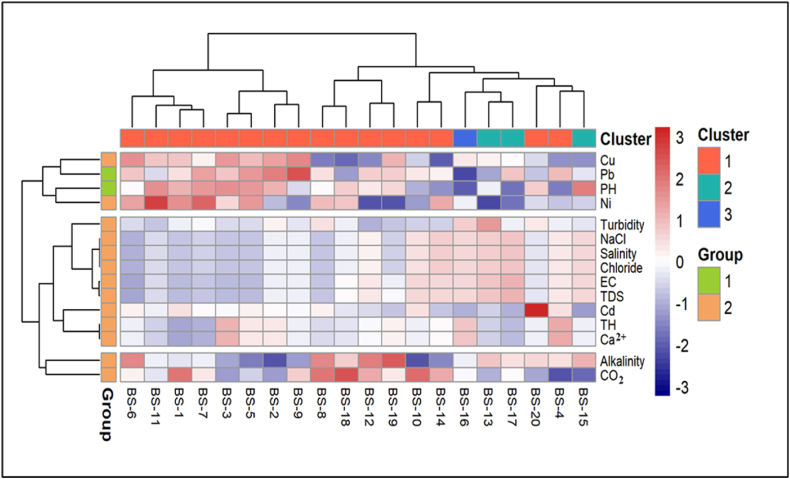
Heatmap clustering of water parameters and locations of investigation area.

This study also included a graphical depiction of data through a heatmap, where individual values are depicted by colors, facilitating a rapid visual summary of complex data. Heatmaps are significant due to their capacity to visually emphasize patterns, facilitating the identification of correlations, outliers, and clusters within the data [115]. In this scenario, the heatmap is paired with hierarchical clustering to illustrate connections between water quality measures and sample groups, with colors ranging from blue (low) to red (high) (Fig. 9). This figure offers a comprehensive examination of water quality, encompassing parameters such as pH, heavy metals (Cu, Pb, Ni), and salinity-related factors (TDS, EC, NaCl). There are three distinct clusters that can be obtained for water parameters from this heat map. The first cluster consists of copper, lead, pH and nickel, indicating contamination of metals and acidity. The second group comprises salinity related factors (TDS, EC), which are also significant with sample stations BS05, BS09 and BS12. The group of third cluster incorporating harness related parameter, which may possibly source of significant mineral concentrations in particular samples (BS 15). The sample locations are grouped into two categories. The first group 1 (BS6) is characterized by decreased contamination levels denotes the pure sources of drinking water. Group 2 comprised various samples exhibiting elevated contamination levels. For instance, BS-5 and BS-12 have elevated concentrations of heavy metals such as Pb and Cd, presumably attributable to industrial contamination. Conversely, BS-9 and BS-15 exhibit elevated salinity and hardness, potentially attributable to seawater intrusion or agricultural runoff [107]. This discovery may assist in identifying primary pollution sources and comprehending water sample contamination, hence enhancing water management and repair efforts.

However, there are various factors that affect the intensity of heavy metals in water which included like geological setting of the water bearing zones, hydrogeological conditions, status of soil, industrial release, water acidity, water oxidation characteristics, and so [103,111]. Hence the different areas water bodies consisted of unlike concentration and nature of heavy metals related contamination [104]. The notable variations have been observed of different parameters in drinking water. Particularly, the distributions of various parameters depend on intrinsic movement and the sources of origin. The drinking water components like salinity, alkalinity, turbidity, EC, TDS, TH, NaCl, Cl**,** all these parameters found as highly concentrated close to the riverside water where might have the possibility of saltwater intrusion [40,74]. It reflects these components originated from the identical source [68]. Furthermore, concentration decreases with distance increases from the region of the riverside. Additionally, the river water quality played a significant role in determining the quality of drinking water in these areas [36,116]. Moreover, the Kirtonkhola river, main source of water supply around Barishal city is now plagued by many types of waste dumping, such as plastic bottles, polythene bags, fertilizer, and chemicals associated with pesticides [74,117]. Rapid urbanization is another significant factor contributing to the depletion of drinking water levels and several forms of water pollution [118]. In contrast, the other parameters such as pH, CO2, and ORP and heavy metals concentration did not follow a trend and varied by regions which mentioned that the groundwater components occurred from different sources [22,45].

### 3.7 Limitations and future implications of this study

The study was conducted in the Barishal City Corporation area of Bangladesh. Consequently, the findings may not apply to other areas with distinct geological attributes. The outcomes may have limited relevance to a broader context. The study was confined to a specific timeframe or a singular sample occurrence. The number of metal analyses was limited due to the shortcomings of the laboratory. However, this is one of the first attempts of its kind within the Barishal city corporation area, where different indices are applied to align with the GIS framework. The future researcher should focus on the increasing number of metal analyses and biological water factors. Data from various times may also be collected to enhance understanding and monitoring of pollution levels, health risks, and water quality. Notwithstanding its constraints, this paper examines environmental concerns and methodological advances in evaluating the contamination and quality of water in the study region. The study's findings can guide policymakers, regulators, and researchers toward more sustainable water management techniques and better safeguards of environmental and human health.

## 4 Conclusions and recommendations

### 4.1 Conclusions

The water's pH indicates slight alkalinity, with high EC, NaCl, and salinity. Although TH, calcium, and chloride concentrations comply with WHO, USEPA, and BDWS guidelines, other indicators raise concerns, with TDS exceeding limits in most samples. Heavy metal concentrations, specifically Cd, Cu, Ni, and Pb, were found to be high, with WHO criteria met by 70 %, 55 %, 100 %, and 65 % of samples, while BDWS acceptable ranges are 95 %, 55 %, 100 %, and 70 %. The total contamination index indicates low to high contamination, while other pollution indices, including the weighted contamination index, show low to medium contamination levels. The PER indicates a high ecological risk for BS 20, with BS1, BS5, and BS7 within a reasonable range and the remaining samples at a moderate level. The hazard score for noncarcinogenic risk was obtained within acceptable limits (<1) throughout this study. The study found that Cd and Ni have the highest variability in cancer risk, with carcinogenic risk exceeding the international safe limit (1E-04). However, children are more vulnerable to carcinogenic and noncarcinogenic risk than adults (male and female). This study observed four categories of water according to WQI, where 55 % of samples are classified into excellent, 10 % are moderate, 15 % are poor, and 20 % are in deplorable conditions. The study found significant positive correlations between EC, TDS, TH, turbidity, salinity, NaCl, chloride, and calcium ions in Barishal City, near the Bay of Bengal. This may be due to slight saltwater intrusion, posing health risks due to its proximity to the coastal region. In the end, this study is a pioneering effort to use a GIS framework with multiple indices to assess groundwater quality in Barishal. Despite limitations, the findings could be beneficial for policymakers and future researchers in sustainable groundwater system development.

### 4.2 Recommendations

To address groundwater pollution challenges and promote sustainable water resource management in Barisal Sadar, this study suggests implementing a continuous groundwater quality monitoring program that focuses on physicochemical and biological parameters and heavy metal concentrations, with GIS-enabled updates to detect contamination hotspots early. To lessen saltwater intrusion, develop strategies or interventions to mitigate it from the Bay of Bengal, as it represents a significant source of salinity and chloride contamination. Furthermore, develop and advocate for rainwater harvesting systems to mitigate dependence on groundwater, particularly in regions with elevated salinity levels. Improving water treatment infrastructure with sophisticated technology to eliminate salt and heavy metals in the water body. Additional studies integrating socio-economic variables are essential to comprehending the extensive effects of groundwater contamination, and collaborative initiatives across academic institutions, governmental agencies, and non-governmental organizations should be enhanced to formulate sustainable strategies. Public health campaigns must enhance awareness of the dangers of heavy metals while facilitating access to alternative drinking water sources, such as desalination units or purification systems, in contaminated areas. By implementing such measures, stakeholders may effectively reduce groundwater contamination, ensure access to safe drinking water, and promote sustainable water resource management in Barisal Sadar and comparable coastal areas.

## CRediT authorship contribution statement

**Md. Numan Hossain:** Writing – review & editing, Writing – original draft, Visualization, Validation, Software, Methodology, Investigation, Formal analysis, Data curation. **M. Farhad Howladar:** Writing – review & editing, Writing – original draft, Supervision, Resources, Methodology, Funding acquisition, Conceptualization. **Sohag Ahammed:** Writing – review & editing, Writing – original draft, Software, Data curation. **Md Rezwanul Haque:** Writing – original draft, Visualization, Software, Methodology, Formal analysis. **Majedul Islam Khan:** Writing – review & editing, Visualization, Methodology, Data curation. **Muyeed Hasan:** Writing – review & editing, Validation, Resources, Formal analysis, Data curation. **Tayabur Rashid Chowdhury:** Writing – review & editing, Visualization, Software, Formal analysis. **Alamgir Hosain:** Writing – review & editing, Resources, Investigation, Data curation.

## Ethical Approval

In this article, none of the authors conducted any studies with humans or animals.

## Availability of date and materials

All data generated or analyzed during this study are included in this published article.

## Declaration of competing interest

The authors declare the following financial interests/personal relationships which may be considered as potential competing interests: Dr. M. Farhad Howladar reports financial support was provided by URC, 10.13039/501100007944SUST. Dr. M. Farhad Howladar reports a relationship with URC, SUST that includes: employment. N/A If there are other authors, they declare that they have no known competing financial interests or personal relationships that could have appeared to influence the work reported in this paper.
